# A New Trade Framework for Global Healthcare R&D

**DOI:** 10.1371/journal.pbio.0020052

**Published:** 2004-02-17

**Authors:** Tim Hubbard, James Love

## Abstract

Current business models for drug development are inefficient and ineffective - drugs are not reaching all who need them. Hubbard and Love contend that it is time to explore some alternatives

The AIDS crisis has brought to public notice what has always been generally true—that the existing business model for drug development leads to high prices and unequal access. There is now widespread dissatisfaction with drug prices in both the developed (Families USA 2003) and developing world (Correa 2000). Governments and health insurers are finding ways to deny access to the newest and priciest products. In the United States and other countries without a universal public health system, the uninsured simply cannot afford the newest medicines. In developing countries, life-saving medicines are priced beyond the reach of most people, a morally offensive outcome (TrueVisionTV 2003). Huge publicity surrounds negotiated price reductions for specific drugs in specific developing countries, yet the effect on the overall access problem is tiny.

Today's high drug prices are a direct consequence of a business model that uses a single payment to cover both the cost of manufacture of a drug and the cost of the research and development (R&D) carried out by manufacturers to discover it. A 20-year patent-based marketing monopoly is then granted to the drug's developers to prevent their prices being undercut by ‘generic’ copies produced by manufacturers who do not have R&D costs to recover. Preventing such ‘free riding’ on R&D has become a global trade issue at the World Trade Organisation (WTO) (Drahos and Braithwaite 2002). The implementation of the TRIPS (Trade-Related Aspects of Intellectual Property Rights) agreement and a growing number of regional and bilateral agreements on intellectual property require most countries to implement tough patent systems that discourage or eliminate competition from manufacturers of generic medicines (Box 1).

Unfortunately, monopoly-based business models have unpleasant side effects. Since the primary responsibility of any company is to maximise return on investment, it is unsurprising that there is pressure on pharmaceutical companies to set drug prices to whatever level gives the highest return, excluding those individuals who cannot afford to pay, rather than maximising the number of patients treated. There is also pressure to misuse the power given by patents, using them as anticompetitive weapons to block innovation and extend marketing monopolies. And there are growing fears that the huge growth in the use of patents is in itself starting to inhibit research (CIPR 2002; Anonymous 2003; Royal Society 2003). Something that is less well recognised is that this system is an enormously inefficient way of purchasing R&D. There is a considerable lack of transparency in pharmaceutical R&D investment, but the available data indicate that only about 10% of drug sales go towards R&D on new products. Only about one-quarter of new drug approvals are rated by the United States Food and Drug Administration (FDA) to have therapeutic benefit over existing treatments (NIHCM 2002; see Figure 1). Measured by investment, only about one-fifth of the 10% is invested in innovative products (Love 2003a). There is also very little research for diseases that primarily afflict the poor (Trouiller et al. 2001; WHO 2003).

**Figure 1:**
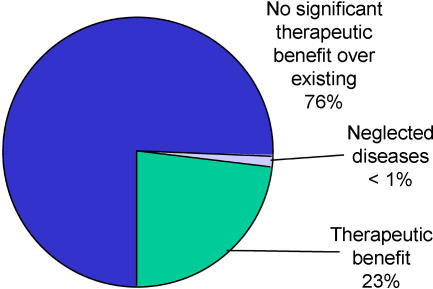
A Breakdown of the 1,035 New Drugs Approved by the FDA between 1989 and 2000 More than three-fourths are classed as having no therapeutic benefit over existing products, so-called ‘me too’ drugs (NIHCM 2002). Less than 1% address diseases that primarily afflict the poor, for which new treatments would have the greatest effect on world healthcare (WHO 2003). Industry trade associations, reports to investors, and data from income tax returns suggest somewhere between 10% and 15% of the $430 billion revenues (reported in 2002) are spent on R&D, but data from regulatory bodies imply that only approximately 2%–3% is actually spent on R&D that leads to new medicines with therapeutic benefits over existing ones, and even this is inflated by research primarily designed to achieve marketing outcomes (Love 2003a; WHO 2003).

Propping up the present structure for financing R&D (Figure 2A) is the widely held belief that the private sector plays a key role in the development of new medicines and that it is necessary to grant patents to incentivise private-sector financing. If this were true, it would make sense to tolerate all sorts of bad outcomes, because the fruits of R&D eventually benefit everyone. But granting a 20-year marketing monopoly on a patented invention is only one way to finance R&D, and the shortcomings of the present system are increasingly hard to ignore. Suggestions for alternatives are beginning to come from many quarters (Baker and Chatani 2002; CGSD 2003; Hubbard and Love 2003; Weisbrod 2003). In this essay, we present practical proposals to modify trade rules based solely on intellectual property so that alternative policy instruments can be used to encourage innovation.

**Figure 2:**
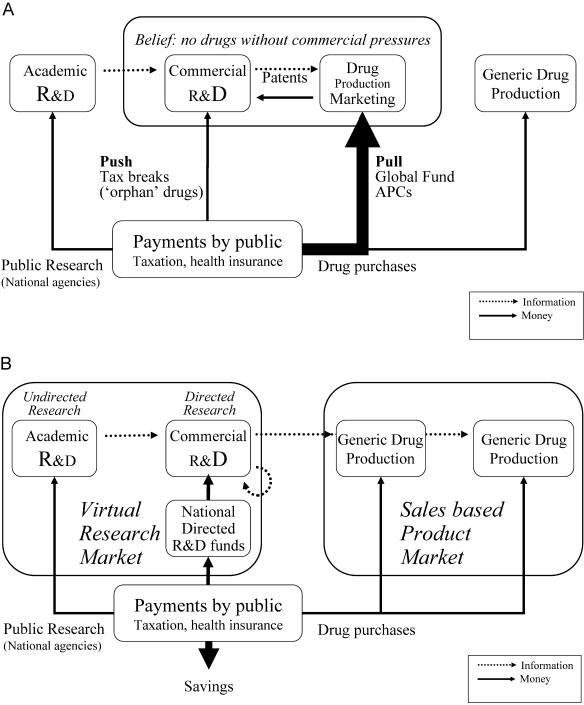
Funding Healthcare R&D (A) A schematic of the way the public currently funds healthcare R&D. Academic research funded by government research agencies is paid for via taxes. This is a mixture of pure research into fundamentals and directed research, including clinical trials. Despite this, there is a dogma that academic R&D cannot produce drugs since it does not have the required commercial pressures to turn ideas into products. Patents ensure the public pays for commercial R&D via their purchase of medicines at high prices, compared to those of generic copies. The distortion of research priorities (too much spent on ‘me too’ drugs and too little on neglected diseases) has been recognised by governments for some time, and a variety of push-and-pull mechanisms have been introduced (or are being considered) to encourage research that more closely reflects public priorities. Examples of push incentives are tax breaks for R&D and other incentives such as special marketing monopolies for products as a reward for investing in research on orphan drugs or testing with pediatric patients. Pull incentives currently being discussed are advance-purchase commitments, with which governments guarantee to buy a certain amount of a drug if one is developed, or prize models. Some of these schemes are thought to be inefficient, particularly those that are indiscriminate and provide expensive subsidies relative to the amount of new R&D they ‘encourage’. (B) A schematic of the way funding of healthcare R&D could work if separate competitive markets for sales and R&D were created. A crucial difference is the absence of monopolies on final products, enabling competition between generic producers and greatly reduced prices. Incentives to develop new drugs would be provided by a new virtual market in R&D. ‘Nationally directed R&D funds’ could represent anything from rewards for innovation using market based mechanisms such as prize models (see text) to centralised funding agencies, similar to the NIH model, or multiple R&D investment funding bodies that compete for new resources. Contributions to R&D could be via taxation or as a legal obligation when paying for private healthcare plans (see text). The ability to design what would be rewarded in the virtual market would allow governments to set R&D priorities and build up local capacity within their own countries. Countries could choose weaker patent protection and create an environment in which all research groups could build on each other's work.

## A New Trade Framework

Analysis of worldwide drug expenditure shows that spending varies, but is close to 1% of the gross domestic product (GDP) in most developed and developing countries (Love 2003b). Assuming that about a tenth of the revenue from the sale of drugs is ploughed back into R&D on new products, that means that countries already indirectly contribute about 0.1% GDP to support this. This contribution is enforced by trade agreements, which require the granting of patents to prevent ‘free riding’ via the purchase of generic drugs (see Box 1). Suppose the World Health Organisation (WHO) developed an R&D contribution ‘norm’ based upon this or a more appropriate figure and that there was international agreement that countries evaluated as meeting this norm would no longer be regarded as ‘free riding’. Trade rules could then be modified to allow countries to meet this norm *by any means*, not just by the implementation of strict TRIPS intellectual property rules, as at present.

Countries that met the norm would then be free to decide whether they wanted to follow a strictly patent-based system as at present, with high drug prices for 20 years, or experiment with new models based on the creation of separate competitive markets for sales and R&D (Figure 2B). Countries adopting the latter system would remove patents on final drug compounds, placing them in the public domain. This would allow them to become a freely traded commodity, creating a competitive manufacture and sales market with low generic prices. At the same time, in order to meet the required R&D contribution norm, they would have to create an efficient R&D virtual market alongside. However, the costs of this would be more than offset by the reduction in drugs prices, making substantial savings for that country overall.

## Business Models for an Effective Virtual R&D Market

The existing system (Figure 2A), despite its failings, does lead to the development of new drugs. The challenge in creating a virtual R&D market is to find viable business models for successful drug development in the absence of marketing monopoly incentives.

One obvious approach is direct funding of drug development. For example, the National Institutes of Health (NIH), the national agency in the United States, already spends $27 billion per year on research, a substantial amount of which is directed towards drug development, including clinical trials. The NIH already has a track record in developing important drugs for severe illnesses, such as cancer or AIDS, showing that this is a viable model. It is also widely recognised that much of the research carried out across the world by similar agencies underpins the existing commercial research that leads to new drugs.

Governments could expand direct funding for drug development, either through the existing structures in academia or through funding R&D arms of existing companies to carry out specific drug R&D. Such directed drug development funding could be similar to existing nonprofit development projects, such as those currently resourced to address treatments for neglected diseases like malaria and tuberculosis (TB). Examples of such projects are the Medicines for Malaria Venture (www.mmv.org), the Global Alliance for TB Drug Development (www.tballiance.org), the International AIDS Vaccine Initiative (www.iavi.org), the Drugs for Neglected Diseases Initiative (Butler 2003b) (www.dndi.org), and the Institute for One World Health (www.oneworldhealth.org).

Many are doubtful that increased direct funding would generate sufficient incentives or be managed efficiently enough. An alternative market-based approach is one in which R&D organisations compete for rewards for specific R&D output, referred to by economists as a prize model (Wright 1983; Kremer 1998; Shavell and van Ypersele 2001). In a simple formulation, governments would place large sums into a fund that would be allocated every year to firms that bring new products to market. This could work with or without patents. If products were protected by patents or other intellectual property claims, the government could grant compulsory licenses (a procedure allowed by trade agreements to override monopoly rights on a patent, in return for compensation to rights owners; see Box 1) and permit rapid introduction of generic competition. The reward system could be a lump-sum payment, eliminating any incentive to continue to market the product, or a long-term payout structure, which would depend upon evidence of both usage and efficacy. Prize systems could be designed to be fairly similar to the current system, with big payoffs for successful entrepreneurs, but even with this approach, there would be huge opportunities to improve welfare. The reward system could be more rational than the existing system, allocating greater rewards for innovative products and less for ‘me too’ products that do not work better than existing products. Premiums could be given for therapies that address treatment gaps or for inventions that pave the way to new classes of drugs.

Organisations competing for prizes might be expected to behave secretly to ensure that they are the ones to obtain ‘credit’ for the fruits of their work. However, progress in research is also driven by free exchange of information. It may be possible to design models that both reward R&D outputs and at the same time encourage complete and continuous openness with intermediate research outputs. There are now a number of examples of open collaborative public goods models (Cukier 2003), such as those used for the Human Genome Project. The proponents of such models point to the success of GNU/Linux in the software field as evidence that major projects can be undertaken with radically different business models. One of the benefits of complete openness is that it allows independent and open evaluation of R&D outputs, which helps in the allocation of ‘credit’ whether in the form or prizes or new research grants. The open-access publishing movement (Brown et al. 2003) has the potential to help in this process by allowing independent analysis of published science, which will help research funding agencies measure research outputs.

## Competitive Intermediators

An R&D contribution norm, established by treaty, would ensure that the amount of money being spent on R&D is maintained. However, new mechanisms would be needed to collect the money to finance the R&D, as it would no longer come via drug sales. This could be via general taxation, although in countries with a private health insurance system this may be anathema. Many will also worry that a centralised national drug development agency taking decisions on R&D priorities and allocation of funds (via prizes or grants as discussed above) could easily become bureaucratic and inefficient.

As a possible alternative, we propose a competitive financing scheme that would work through R&D investment intermediators. These R&D funds would be licensed and regulated (like pension funds). Their role would be to manage R&D assets on behalf of consumers. Individuals (or employers) would be required to make minimum contributions into R&D funds, much as there are mandatory contributions to social security or health insurance or to pension funds. Government would set the required contribution, but the individual (or employer) would be free to choose the particular intermediator that received their contributions. Intermediators would compete to attract funds to invest in R&D on the basis of their prowess for drug development and upon their priorities. Different business models for financing R&D could be tested in such a market, with intermediators experimenting with prize systems, direct investments in profit or nonprofit entities, open collaborative public good models, or other approaches.

## A Change for the Common Good

We believe the economics of a change in the paradigm for funding R&D are highly favourable. Taken together, the two core steps of changing the trade framework and moving away from marketing monopolies can change the world in a positive way. We can raise global R&D levels as a matter of policy and ensure that resources flow into the areas of the greatest need, and we can do so knowing that the poor and the rich will have access to new inventions at marginal cost. Policy-makers will be weaned from their current unhealthy addiction to ever-higher levels of intellectual property rights as the only instrument to raise R&D levels, a path that has increasingly reached diminishing returns or become counterproductive. With new instruments to address the overall levels of R&D investment, policy-makers can more constructively address the well-known inefficiencies in the patent system without the fear that global R&D levels will suffer and explore alternative models (Butler 2003a). At the same time, the system of prescribing medicines will be transformed by a substantial reduction in the distorting influences of the current multibillion-dollar industry of marketing medicines to doctors and (increasingly) directly to the public. Similarly, without marketing monopolies to protect, there will be far less spent to influence the governments that set the rules that regulate such monopolies. If implemented worldwide, one of our most vexing ethical dilemmas can be resolved in a manner that actually promotes the Doha Declaration on TRIPS and Public Health mandate to encourage access to medicine for all.

##

**Box 1. Trade Agreements on Intellectual Property**

The most important is the World Trade Organisation (WTO) agreement on Trade-Related Aspects of Intellectual Property (TRIPS), which requires member countries to issue 20-year patents on all fields of technology. All but the least-developed countries must comply by 2005. Going much further than the TRIPS are a plethora of regional and bilateral ‘TRIPS-Plus’ trade agreements, pushed in particular by the United States, which require even higher levels of intellectual property protection, such as limitations on the use of compulsory licensing, a tool used by governments to override the strong exclusive rights of a patent in return for compensation to patent owners. In 2001 the WTO adopted the Doha Declaration on TRIPS and Public Health, which said that ‘the Agreement can and should be interpreted and implemented in a manner supportive of WTO Members’ right to protect public health and, in particular, to promote access to medicines for all'. In order to promote ‘access to medicines for all’, countries have to find new ways of financing R&D.

## Abbreviations

FDA: Food and Drug Administration
GDP: gross domestic product
NIH: National Institutes of Health
R&D: research and development
TB: tuberculosis
TRIPS: Trade-Related Aspects of Intellectual Property
WHO: World Health Organisation
WTO: World Trade Organisation
